# Microgravity, Stem Cells, and Cancer: A New Hope for Cancer Treatment

**DOI:** 10.1155/2021/5566872

**Published:** 2021-04-29

**Authors:** Uğur Topal, Cihan Zamur

**Affiliations:** ^1^Department of Molecular Oncology Health Sciences Institute, Health Sciences University, Istanbul, Turkey; ^2^Department of Pediatrics, University of Health Sciences, Sariyer Etfal Training and Research Hospital, Istanbul, Turkey

## Abstract

Humans are integrated with the environment where they live. Gravitational force plays an important role in shaping the universe, lives, and even cellular biological processes. Research in the last 40 years has shown how exposure to microgravity changes biological processes. Microgravity has been shown to have significant effects on cellular proliferation, invasion, apoptosis, migration, and gene expression, specifically in tumor cells, and these effects may also exist in stem and cancer stem cells. It has also been shown that microgravity changes the effects of chemotherapeutic drugs. Although studies have been carried out in a simulated microgravity environment in cell culture lines, there are few animal experiments or true microgravity studies. Cancer remains one of the most significant problems worldwide. Despite advances in medical science, no definitive strategies have been found for the prevention of cancer formation or to inform treatment. Thus, the microgravity environment is a potential new therapeutic strategy for future cancer treatment. This review will focus on current knowledge on the impact of the microgravity environment on cancer cells, stem cells, and the biological behavior of cancer stem cells.

## 1. Introduction

Gravity is a universal power with an impact on all life forms existing on earth and the biological processes contained within them. Physical forces, such as gravity and electromagnetism, played a part in shaping the evolution of life on earth, and they continue to influence living processes in organisms. In the last four decades, the development of the space industry has led to new discoveries regarding the effects of microgravity (*μg*) on biological life and processes. It has been shown that microgravity can alter important properties of cells including cell morphology, proliferation, and migration [1, 2]. Gravitational biology and its effect on cancer cells are of great interest and remain a current topic in space research.

Studies can be carried out in a microgravity environment on the International Space Station (ISS), which provides long-term cell culture in true *μg*. However, it is difficult to distinguish the effects of microgravity in space from those of cosmic radiation [3]. In addition, research on the ISS is very costly. For these reasons, studies are carried out using world-simulated microgravity (s‐*μg*) platforms. To conduct stem cell research in simulated microgravity conditions, platforms and devices comprised of the rotating wall vessel (RWV), custom-made random positioning machine (RPM), rotary cell culture system (RCCS), 2D clinostat, and 3D clinostat can be used [4–7].

s-*μg* is different from real *μg*, and differently designed devices are used in each type of study. When comparing results from r- and s-*μg*, it should be noted that there are fundamental differences between the two conditions. Cell culture in r-*μg* in space takes place in an essentially force-free environment without perturbations of the culture medium except for the naturally occurring diffusion of nutrients and cellular waste products due to local concentration gradients. However, cells on an s-*μg* device, such as an RPM, RWV, RCSS, or CN, experience residual acceleration depending on their distance from the center of rotation, shear forces, and a constant mixture of the cell culture medium. Although these effects are not necessarily detrimental, they introduce additional factors and variations over the course of the cell culture procedure, which can eventually lead to deviations between results from r- and s-*μg* experiments [5, 8].

Significant differences can also occur between various s-*μg* methods. The RWV is a horizontally rotating vessel with no internal mechanical agitator. A central silicone membrane is present in the RWV, which delivers oxygen via diffusion, avoiding the production of bubbles that are disruptive to the growing cells. The vessel is completely filled with culture media and thus has no air-liquid interface. Because there are no internal moving parts, the vessel provides a culture environment that is characterized by low shear and low turbulence. Within the RWV, adherent cells are grown on microcarrier beads to provide a solid support; the cells readily attach and cover the surface of the microcarriers. With continued growth, multiple cell-covered beads coalesce, undergo cellular bridging, and generate high-density 3D aggregate structures. This occurs under conditions of optimized suspension and uniform mixing to allow for the effective transfer of nutrients and metabolic waste [9, 10].

The random positioning machine (RPM; also known as the 3D clinostat) can support certain conditions of the space microgravity environment, including lack of sedimentation, to facilitate cell colocation and growth of multicellular spheroids. As opposed to the RWV in which cells are cultured entirely in suspension, the RPM is carried out in a tissue culture flask containing a subconfluent monolayer of cells that is affixed to the center of a platform in an interconnected framework comprised of two perpendicular arms that rotate independently of each other. This creates a continuous random directional adjustment of the culture flask. The flask is filled completely with media, and aeration occurs via a gas-permeable cap. During growth in the RPM, cells can detach from the surface of the flask and form multicellular spheroids. Therefore, incubation in the RPM yields cell growth in suspension as well as in cells remaining adhered in 2D culture. In the RPM, the gravity vector is constantly reoriented, as occurring in clinorotation, but this occurs with increased directional randomization in a manner such that no prevailing orientation occurs [9, 11].

Each of the model systems offers operational advantages and constraints that can be used to approach experimental problems. For example, large constructs are difficult to maintain in suspension in the RWV; hence, 3D growth can become size limited. However, because a small amount of fluid shear is present in the RWV; this system can be used to study the influence of shear stress on cancer cell constructs, as well as the RPM.

## 2. The Effect of Microgravity on the Biological Behavior of the Cancer Cell

Many studies have demonstrated the suppressive effect of microgravity on the viability and growth of cancer cells. In one study, microgravity significantly reduced the activity of cells, and this effect was directly proportional to the duration of microgravity [12].

BCL-2, an antiapoptotic protein—p53 being one of the proteins associated with the inhibition and apoptosis of BNIP3—was shown in thyroid cancer cells in the microgravity environment where the simulation of induction related to PARP and BAX had been done [13]. The p53 protein downregulates proteins such as MDM2, p21, and BAX, which regulate apoptosis and cell cycle in a microgravity environment [14]. In one study, Zhao et al. demonstrated that simulated microgravity induced apoptosis through upregulation of caspase-3, caspase-7, and caspase-8, resulting in apoptosis via downregulation of molecules regulating the NF-*κ*B pathway, including TICAM, UEV1A, TRAF2, and TRAF6 [15]. The CAV1 protein is sensitive to gravity and regulates cellular differentiation, proliferation, and apoptosis. Studies have shown that CAV1 is decreased in cancer cells after 72 h in a microgravity environment [16]. Based on these studies, we can conclude that microgravity affects cancer cells via inhibition of survival signaling pathways and induction of programmed cell death.

Studies have also found that proteins regulating the cell cycle, such as cyclin D1 and B1, are downregulated in a simulated microgravity environment in breast and colorectal cancer cell lines [12, 13]. The ATR/ATM and CDK 1/2 proteins are required for the cell cycle transition from S to G2, and these proteins are decreased under microgravity. This was demonstrated by flow cytometric analysis showing a decrease in the amount of cancer cells in the G2 phase [15]. The effect of the PCNA-CDK-cyclin protein complex, which is required for cell cycle progression, was reduced under simulated microgravity conditions. Simulated microgravity functions by inducing the expression of the p21 protein, which then inhibits DNA replication and the cell cycle. In addition, p21 forms complexes with PCNA that inhibit CDK activity. This interaction prevents the phosphorylation of RB proteins and the release of the transcription factors which bind to it, resulting in a blockade at the G1 phase checkpoint [17].

On earth, a true microgravity environment may be created by a free fall from a drop tower. However, the duration of microgravity obtained by these methods is generally too short for cell cultivation. Thus, clinostat, which provides a long-term taSMG environment, has been applied in studies of the effects of microgravity on cells. Time-averaged simulated microgravity (taSMG) has been proposed, wherein a continuous change in the direction of gravity enables simulation of the effect of microgravity on cells. Kim et al. examined the impact of microgravity on Hodgkin lymphoma cells (L-540) compared to normal human dermal fibroblast cells. The L-540 and HDLM-2 human lymphoma cell lines were cultivated in a 3D clinostat with constant angular velocities. The angular velocities used were determined via an optimization process to provide evenly distributed gravity. Cells were seeded in 25 cm^2^ flasks with a total of 5 × 10^6^ cells. Before being placed in the clinostat, the flasks were carefully filled with medium (approximately 80 ml) without air bubbles to avoid shearing of the fluid. After the flasks were fixed on the stage of the clinostat, the clinostat was operated for 1, 2, and 3 days in a commercially available incubator set at 37°C and supplied with 5% CO_2_. The control cells were grown in parallel at 1g and kept statically in the same incubator as the clinostat. The same procedure was repeated four times. Results showed that microgravity halted lymphoma cancer cell proliferation and induced cell death, where the normal cells were not affected [18]. Colony formation studies in melanoma, colorectal, and leukemia cell lines have demonstrated that microgravity reduces the cancer cell's ability to form colonies [13, 15]. It is possible that low gravity affects the genes and proteins that control the cell cycle, thus preventing cancer cells from producing colonies.

Chang et al. showed a reduction in migration in the A549 lung cancer cell line after 24 h of exposure to microgravity conditions. The expression of matrix metalloproteinase-2 (MMP2) and antigen MKI67 was also decreased compared to cells grown in normal gravity [19]. Microgravity environments also increase the expression of TIMP genes, causing downregulation of MMP genes and reducing cell migration [20]. Cancer cell migration causes metastasis, and epithelial mesenchymal transition (EMT) is an event which plays an important role in the cancer metastasis process. Vimentin is an important regulatory factor of EMT. Vimentin is an important marker of EMT and a necessary regulatory factor for the migration of mesenchymal cells, whose activity is interrelated with E-cadherin expression. Previous studies have shown that decreased function of E-cadherin could result in aggressiveness, dedifferentiation, and metastasis in many carcinomas. The available evidence suggests that microgravity can rearrange vimentin and form nucleo-vimentin polymers. This alteration can reduce the number of vimentin molecules that exert physiological effects, leading to altered migration of cancer cells. Furthermore, EMT is also involved in other steps in the cancer process. Under microgravity, the TGF-*β* signaling pathway simultaneously inhibits the expression of two EMT-induced genes, *snail1* and *snail2*, and promotes apoptosis [20, 21].

In a study using space microgravity, Li et al. showed that space microgravity suppressed glucose metabolism, modulated the expression of cellular adhesive molecules such as ICAM-1, VCAM-1, and CD44, and depressed proangiogenic and proinflammatory cytokine secretion. Microgravity also induced the depolymerization of actin filaments and microtubules, promoted vimentin accumulation, restrained collagen I and fibronectin deposition, regulated mechanotransduction through the focal adhesion kinase and Rho GTPases, and enhanced exosome-mediated mRNA transfer. After the long-term spaceflight in this study, vimentin accumulated in endothelial cells (ECs), especially in the perinuclear region, which might compensate for the loss of F-actin and microtubules [22].

The organization of microtubules in a particular model depends on gravity, and these processes are blocked under microgravity conditions. True and simulated microgravity activates various signaling pathways, leading to the abnormal expression of various adhesion molecules, which in turn can lead to changes in the 3-dimensional structure [13]. In their microgravity study of MCF-7 (human breast adenocarcinoma cells), Kopp et al. found disruption of microtubules in MCF-7 cells within 4 h after exposure to induced microgravity [23]. The F-actin cytoskeleton plays a part in certain changes in signaling pathways, along with changes in cell morphology and other functions under microgravity. Boonstra found that the amount of F-actin increased for 7 min in A431 epidermoid carcinoma cells in a true microgravity environment [24]. This result suggests that the actin microfilament structure responds to changes in gravity and that the building of the cytoskeleton can be affected by microgravity.

Lewis et al. investigated the effect of microgravity on the microtubule cytoskeleton of cells from a human lymphoblastoid cell line. Results showed that microgravity caused the filaments to shorten and join together, with no normal branching in the cell membrane [25]. Microgravity disrupts the intracellular tension balance and causes irregular cytoskeleton formation [26].

## 3. The Effect of Microgravity on the Biological Behavior of Stem Cells

Embryonic stem cells (ESCs) are a type of stem cell isolated from embryos or a primitive gonad. ESCs have infinite proliferation capacity, self-renewal, and versatile differentiation *in vitro*. ESCs can be induced to differentiate into any type of cell *in vivo* and *in vitro*. Therefore, ESCs can be used as a cell model in a microgravity environment [27].

Wang et al. used a three-dimensional (3D) clinostat to study mouse embryonic stem cells (mESCs). They demonstrated that the total amount of mESCs cultured under simulated microgravity was significantly reduced compared to mESCs cultured under normal gravity. However, they found no significant difference in the cell cycle. Microgravity did not have a damaging effect on DNA, but it prevented the repair of DNA damaged by radiation. They also investigated whether SMG could affect the repair of radiation-induced DNA lesions of mouse embryonic stem cells (mesx). Mouse ES cells exposed to 8 Gy gamma-ray radiation (IR+) or not exposed (IR-) were incubated for 0 h, 1 h, and 2 h under 1g or s-*μg* conditions. A neutral comet assay was performed to measure DNA double-strand breaks (DSB) induced by ionizing radiation, and the progression of DSB repair is indicated by the loss of the comet tail. At 0 h after radiation, as expected, the tail moment was significantly longer than that at the other time points, indicating that the DNA damage induced by gamma-ray radiation was gradually repaired over time. However, the progression of repair was different under different gravity conditions. At 1 h following radiation, the tail moment of cells cultured in s-*μg* was significantly longer than that of cells in 1g. At 2 h, there was also a difference in tail moment between the two groups of cells, though it was not significant. This indicates that SMG can impair the early-stage repair of radiation-induced DNA lesions of mES cells. Overall, this study showed that stem cells are sensitive to microgravity [28].

In a study examining the development and formation of embryoid bodies from mESCs under microgravity, Liu et al. found that ESCs rapidly formed large numbers of embryoid bodies accompanied by different types of cellular differentiation under simulated microgravity. This finding leads us to conclude that microgravity has stimulating effects on the differentiation of ESCs [29].

Mesenchymal stem cells (MSCs) are an important member of the stem cell family, and they can be found in most postnatal tissues and organs. The effect of microgravity on the morphology and cytoskeleton of MSCs can be clearly seen. In a simulated microgravity environment, the attachment of the cytoskeleton and MSCs changes dramatically.

In a long-term (20 days) simulated microgravity study on human MSCs, Gershovich and Buravkova found that hMSCs cultured under microgravity had a disrupted actin cytoskeleton, redistributed vinculin, and increased integrin a2 expression. In addition, the number of cells expressing vascular cell adhesion molecule 1 (VCAM-1) was increased. This outcome suggested that microgravity can cause a change in microfilaments and the adhesion of MSCs [30].

Dai et al. found that the proliferation of hMSCs cultured on a gyroscope was inhibited, and their cell cycle was halted in the G0/G1 phase [31]. An experiment conducted in space during the KUBIK space mission ISS 12S with hMSCs cultured confirmed this finding. It has been suggested that decreased proliferation potential of hMSCs results from decreased expression of cell cycle genes [32].

In another study, Huang et al. identified gravity as a powerful factor which can highly influence the differentiation of MSCs. They found that while microgravity can cause MSCs to differentiate into force-insensitive cells such as adipocytes, with an increased gravity force, MSCs can differentiate into force-sensitive cells such as osteoblasts and myocardial cells. Without doubt, this outcome has significant potential for advancing tissue engineering, regenerative medicine, and stem cell-based therapy [33].

Chen et al. examined the neural differentiation of MSCs under microgravity conditions and found that in cells cultured in a neural differentiation environment under microgravity conditions, secretion of neurotrophins such as tyrosine hydroxylase (TH) and choline acetyltransferase (CHAT), microtubule-associated protein 2 (MAP-2), nerve growth factor (NGF), brain-derived neurotrophic factor (BDNF), and ciliary neurotrophic factor (CNTF) increased. The above results indicate the stimulating effect that microgravity has on the neural differentiation potential of MSCs [34].

Hematopoietic stem cells (HSCs) are pluripotent and self-regenerating cells in the bone marrow that express the membrane-bound surface antigen CD34. It has been shown that microgravity can significantly inhibit cell turnover and migration of bone marrow CD34+ cells. CD34+ cells cultured under a simulated microgravity environment exit the G0/G1 phase of the cell cycle at a slower rate compared to cells cultured under normal gravity [35]. Long et al. conducted a study using human erythroleukemia cells (K562) cultured in RCCS and discovered that microgravity prevented the proliferation of K562 cells and halted the cell cycle in the G0/G1 phase [36]. Over a period of 11–13 days during the Space Shuttle Missions STS-63 (Discovery) and STS-69 (Endeavor), the total number of cultured cells increased less than the ground controls [37]. From these studies, we can conclude that microgravity inhibits the proliferation and differentiation of hematopoietic precursors.

## 4. The Effect of Microgravity on the Biological Behavior of Cancer Stem Cells

Numerous publications report information regarding how cancer cells behave in a microgravity environment, but there is limited information on the behavior of cancer stem cells (CSCs) in a microgravity environment. CSCs represent a subset of cells within liquid and solid tumors. The term CSC was first introduced 25 years ago by Dr. John E. Dick and colleagues. CSCs have the characteristics of somatic stem cells and exhibit asymmetric division, self-renewal, and differentiation properties. CSCs can replicate the parent tumor by being transplanted into a host and play an important role in tumor pharmacological resistance. CSCs are beginning to be considered the primary therapeutic target for cancer treatment due to their properties [7, 38, 39].

The heterogeneity of tumor cells and the presence of CSCs are associated with a poor prognosis in cancer patients. Cancer cells are divided into two phenotypes in the microgravity environment. Some grow by sticking to the bottom of the cell culture flasks, and the others assemble into 3D spheroids. Multicellular spheroids (MCS) possess the characteristics of the primary tumor and also exhibit root-like properties. Therefore, they make an excellent model for detailed CSC research [7, 40].

As with most tumors, lung CSCs display increased CD44 and CD133 cell surface markers. Pisanu et al. subjected lung CSCs enriched with the stable non-small-cell lung cancer cell line H460 to microgravity. The study found that lung CSCs, when exposed to low gravity, are resistant to selective differentiation. In addition, the apoptosis of CSCs increased in comparison to those grown under normal gravity. Therefore, when lung CSCs are cultured in microgravity, similar to somatic stem cells, they lose their stem cell properties. Another report consistent with this observation was that the Nanog and Oct4 genes were downregulated in addition to a decrease in ALDH levels [16].

Little is known about how gastrointestinal stem cells behave in a microgravity environment. Arun et al. cultured CSCs and HCT116 colon cancer cells in a rotary cell culture system (RCCS) and found that the percentage of CSCs expressing CD44 and CD133 simultaneously increased within the cell population. They also showed that microgravity affected the growth/differentiation control elements PTEN/FOXO3/AKT. Also, low gravity increased autophagy and increased the number of giant cancer cells harboring the full nuclear localization of Yes-Associated Protein (YAP) [41].

Kelly et al. investigated human osteosarcoma cells (SAOS-2 cells) exposed to a hydro focus bioreactor (HFB) and rotary cell culture system (RCCS) developed by NASA, and they found that CSCs were stimulated to proliferate when cultured in microgravity media. In addition, microgravity functioned by sensitizing CSCs to chemotherapeutic agents. Additionally, various cell types such as prostate cancer cells, osteosarcoma cells (SAOS-2), lung cancer cells, and melanoma cells were investigated by the authors on the HFB system. Results showed that exposure to HFB increased CD133-positive cell growth in the various cell lines [42].

There are only a few published studies investigating the effect of simulated microgravity on CSCs. Results in this area depend on the type of cells and the choice of microgravity simulator. It has also been found to be a very good method for successfully enriching CD133-positive CSCs in several cancer cell lines. Due to the different results achieved when using RPM, RCCS, or HFB devices, more research on different cancer types is required to fully study cancer stem cell biology [5].

## 5. Studies of the Effect of Microgravity on Cancer Cell Lines

Prasanth et al. exposed erythroleukemic and leukemic cancer cells to microgravity with RCCS for a duration of 48 h. They discovered that when leukemic cancer cells were treated with daunorubicin, they showed increased chemotactic migration (*p* < 0.01) following simulated microgravity (s-*μg*) compared to the normal gravity on earth (1g). This suggests that microgravity affects the response of cancer cells to chemotherapy in a drug-dependent manner [43]. There are few publications regarding the effect of chemotherapeutic drugs in a microgravity environment, which is still an open discussion with many areas for improvement.

To evaluate the effect of stimulated microgravity on the growth of glioma, Deng et al. studied U251 cells under low gravity for different time periods. Cell proliferation was measured via the CCK8 assay, and results showed that s-*μg* inhibited the activity of U251 cells in a time-dependent manner. The longer the microgravity environment persisted, the lower the activity of the U251 cells. Microgravity for 48 to 96 h significantly stimulated cell death in U251 cells, and cell activity was decreased to approximately 45% [44].

Vidyasekar et al. studied the DLD1 colorectal cancer cell line and the MOLT-4 lymphoblast leukemic cell line under microgravity (RCCS) and found a decrease in colony-forming ability and cell viability. They also found dysregulation in oncogenes, cell cycle genes, and progression markers, such as MIR17HG, MIR22HG, MIR21HG, CD44, JUNB, MYC, and CD117 [13].

Chung et al. conducted a study using the H1703 human squamous carcinoma cell line and the A549 human lung cancer cell line via the clinostat-simulated microgravity environment. They found that different cell lines exhibited different behaviors in a microgravity environment. While the simulated microgravity medium did not significantly affect cell proliferation in the A549 cell line, proliferation was inhibited in the H1703 cell line. Increased migration of both the H1703 and A549 cell lines was detected after exposure to simulated microgravity compared to the control group under normal gravity. Although they could not fully explain this difference, they hypothesized that it might be due to the tumor microenvironment [45].

Ma and Pietsch used a short-term parabolic flight and the long-term Shenzhou 8 space mission for true microgravity and RPM for simulated microgravity to study a follicular carcinoma cell line (FTC-133). Their study evaluated the differences between simulated and real microgravity. Results revealed changes in the expression of genes responsible for the cytoskeleton, apoptosis, adhesion/extracellular matrix, migration, proliferation, angiogenesis, and signal transduction. Proteins and genes involved in the regulation of the proliferation and metastasis of cancer cells are similarly regulated under RPM and spaceflight conditions. The resulting effect shifted the cells toward a less aggressive phenotype. During parabolic flight, gene expression is usually reversed. With these studies, Ma and Pietsch concluded that simulated microgravity methods can mimic real microgravity and that studies can be performed in these systems [46].

Results from microgravity research can be used to rethink conventional cancer research and may help to pinpoint the cellular changes that cause cancer. They supported this view [47]. FTC-133 cells grown on the RPM showed higher levels of NF*κ*B p65 protein and apoptosis than controls grown under normal gravity (1g), a result which was also found earlier in endothelial cells [48]. Bauer et al. [49] detected 69 proteins that significantly accumulated in thyroid cancer cells in the transition from 2D cell growth in normal gravity to spheroid growth in *μg*. Using pharmacological targeting or silencing of individual genes, they inhibited selected signaling pathways. MCF-7 breast cancer cells incubated with the NF*κ*B inhibitor dexamethasone showed dose-dependent inhibition of spheroid formation [50]. The artificial glucocorticoid dexamethasone functionally inhibits NF*κ*B and NF*κ*B-dependent gene expression. These *μg* research results prove that NF*κ*B plays a crucial role in spheroid formation in tumors.

Shi et al. studied the effect of microgravity on the invasion and migration potential of glioblastoma cells in a 2D-clinostat microgravity model using U87 human glioblastoma cells. They found that modeled microgravity stimulation significantly attenuated invasion and migration potential, decreased thapsigargin- (TG-) induced store-operated calcium entry (SOCE), and downregulated the expression of Orai1 in U87 cells. Inhibition of SOCE by 2-APB or stromal interaction molecule 1 (STIM1) downregulation mimicked the effects of microgravity on the invasion and migration potential of U87 cells. Furthermore, upregulation of Orai1 significantly weakened the effects of MMG on invasion and migration potential in U87 cells. Therefore, these findings indicated that modeled microgravity stimulation inhibited the invasion and migration potential of U87 cells by downregulating the expression of Orai1 and sequentially decreasing the SOCE. This suggests that modeled microgravity might be a new potential therapeutic strategy in glioblastoma treatment [51].

Taga et al. used murine B16-F10 melanoma cells in their work using a rotating-wall vessel bioreactor microgravity environment. Results showed a decrease in proliferation and an increase in the percentage of apoptotic cells and melanin production. When they examined the tumor-forming ability of melanoma cells cultured in a microgravity environment and melanoma cells cultured in a normal gravity environment, they concluded that the microgravity environment was advantageous to tumor formation [52].

Morabito et al. found a decrease in proliferation and a delay in cell cycle progression in the human TCam-2 cell line after 24 h of simulated microgravity. Increased intracellular Ca^2+^ levels and reactive oxygen species were associated with an increase in anaerobic metabolism. Interestingly, all these events were transient and disappeared after 48 h of exposure. In the TCam-2 cell model, simulated microgravity activated oxidative mechanisms, causing a decrease in proliferation and transient events such as autophagy activation [53]. With these studies, they showed that the effects of microgravity can vary depending on the duration of exposure.

Chen et al. cultured HGC-27 stomach cancer cells in the RCCS bioreactor. Then, they used liquid chromatography-mass spectrometry to study the effects of simulated microgravity (s-*μg*) on the metabolism of HGC-27 cells. Compared to the normal gravity group, phosphatidyl choline, phosphatidyl ethanolamine, arachidonic acid, and sphinganine were significantly upregulated under s-*μg* conditions, while phosphatidyl serine, sphingomyelin, phosphatidic acid, creatine, L-proline, pantothenic acid, adenosine triphosphate, oxidized glutathione, and adenosine triphosphate were significantly downregulated. This study demonstrated changes in the metabolic expression of HGC-27 gastric cancer cells in a microgravity environment [54].

## 6. Microgravity and the Immune Response

Microgravity causes changes in both innate and adaptive immune systems, leading to changes in immune responses. The effect of microgravity on the immune system has been investigated in various studies. In the Soyuz, Skylab, Salyut, and Space Shuttle programs, a decrease in immune cell function was observed after varying flight times [55]. A 50% reduction in lymphocytic response after mission compared to the preflight response was also found in 12-month studies conducted by Russia at the Mir space station [56].

Kopp et al. demonstrated overexpression of the inflammatory factors IL-6, IL-7, and IL-17 in thyroid cancer cell lines studied under microgravity conditions. In addition to these molecules, they found that the proteins activated by proinflammatory cytokines were upregulated in cancer cells [57].

Studies on the effect of microgravity on immune cells in mice have shown suppression of the mitogenesis of lymph cells and the activity of cytotoxic T cells (CTC). It was also found that T cell percentages, numbers, response to a strong mitogen, and secretion of cytokines, which are critical for an optimal immune defense and homeostasis, were significantly affected [58, 59].

Bradley et al. conducted a study using murine JAWS II DC and E.G7-OVA cultures in a simulated microgravity environment. They found that regarding activation signaling for cytokine production and recognition of cytotoxin release, the interaction of the CD8+ T cell TCR with peptide/MHC I was increased [60].

Shi et al. conducted a study using mouse hematopoietic stem and progenitor cells (the Tianzhou 1 cargo ship program) under simulated microgravity and demonstrated that microgravity had a significant reduction effect on macrophage differentiation, decreased the number of macrophages and functional polarization, and caused changes in gene expression profiles [61].

Li et al. exposed mesenchymal stem cells (MSCs) to simulated microgravity and then injected them into nude mice as an anticancer vaccine. The simulated microgravity MSCs showed an increase in the expression of the MHC1 and HSP proteins. Th1, which completely inhibited the proliferation of A549 human lung cancer cell lines and reduced tumor size and weight, induced cytokine and CD8-mediated cytotoxic responses and promoted the apoptosis of tumor tissue [62].

## 7. Therapeutic Approaches

Tumor cells behave differently under varying gravity conditions. These changes include cell cytoskeleton rearrangement, aggregation, cell cycle arrest, apoptosis, and migration. This can be considered an orientation toward a less aggressive phenotype. Microgravity-based studies are conducted *in vitro*, and limited evidence exists from human or animal experiments. The most critical question is regarding the safety of the microgravity strategy in humans. Another problem is related to the tools that can be used to create a microgravity environment in the body. Recent discoveries of nanomagnetic fluids have opened new avenues in cancer treatment. Various new methods have been developed to use this magnetic fluid to expose tumor cells to microgravity. It is thought that the magnetic fluid model of microgravity may be suitable for clinical applications in the future [12, 20, 21, 63].

It has been found that resistance to chemotherapeutic drugs is reduced in tumor cell lines in microgravity environments, but this has been tested in a limited number of tumors and with only a few chemotherapeutics [43]. Currently, there are no studies in the literature examining the effect of radiotherapy in true and simulated microgravity environments.

Cancer treatment and the immune system have a close relationship. Most immune response disorders can lead to cancer formation or cause resistance to therapy. Some studies have confirmed the effects of microgravity on immune cell activation, proinflammatory and inflammatory protein accumulation, and induction of cytokine secretion [64, 65]. Based on this, we can conclude that microgravity environments are a potential tool for cancer treatment that exert an immunomodulatory effect.

Unexpectedly, there are also studies showing that microgravity had a positive effect on cancer cells. In human lung cancer cell lines, migration increased following exposure to microgravity [45]. Researchers have found some evidence to suggest that microgravity can cause some types of cancer. For example, the protooncogene MYC was shown to be upregulated in colorectal cancer and downregulated in leukemia. CD117, another protooncogene, was upregulated in leukemia and downregulated in colorectal cancer [13].

## 8. Conclusions and Perspectives for the Future

Technological developments have resulted in new dimensions in cancer treatment studies. Cancer studies continue to be done in low-gravity environments, where targeted therapies and cancer stem cell studies are frequently carried out. The development of potential drugs to target proteins affected by microgravity also shows pharmacological potential. Altered gravity conditions provide a new technology that helps in detecting changes in proteins that may be new targets for cancer drug development. A large number of proteins which may serve as promising targets and available drugs have been identified. For example, studies have shown that daidzein targets caveolin-1, camptothecin targets the ubiquitin-like protein ISG15, dexamethasone and BAY 11-7082 target NF*κ*B p65, MT189 targets paxillin, baicalein targets ezrin, and curcumin targets HMOX-1. The conduct of controlled studies in microgravity can further enhance our understanding of the fundamental role of biophysical forces in cancer cell growth and function. The nature and treatment of cancer are limited to the knowledge of human beings. Cancer biology has been described in the environmental conditions we can observe. The uncertainties about cancer cells, whose biological behavior we are trying to define in different physical environments, should be more easily resolved with advancing technology. Future creation of a microgravity environment would be an important force in cancer treatment. Treatment centers that can establish a microgravity environment could bring hope to desperate cancer patients. Finally, microgravity research in space and on earth can be used to support the development of treatments that are patient-specific and bring forth novel ideas for cancer research and regenerative medicine. Humankind has traveled in search of the unknown, but our knowledge can be compared to the information on one page of a book in a giant library. Imagination is needed for the realization of new discoveries.

## Data Availability

The quantitative and qualitative data supporting this systematic review are from previously reported studies and datasets which have been cited. These prior studies (and datasets) are cited at relevant places within the text as reference.
